# Analectic

**Published:** 1837

**Authors:** 


### MISCELLANEOUS INTELLIGENCE.

### ANALECTIC, ANALYTICAL, AND ORIGINAL.

### 1.—ANALECTIC.

#### AD SYLVAS NUNCIUS.

1. *Some experiments and observations on tying the carotid and vertebral arteries, and the pneumogastric, phrenic, and sympathetic nerves.* By Sir Astley Cooper, Bart.—The anastomosis of arteries in all parts of the animal frame, and the circuitous channels through which the blood, when arrested in its progress along a principal trunk, is conveyed to its destination, have been for some time well ascertained; and the advantages arising from this arrangement of vessels in the natural condition of the body, as well as the safety afforded by it under certain accidents, diseases, and operations, are perfectly understood.

The existence of these anastomising vessels has been proved, by the examination of diseases in which blood-vessels have been obliterated, by experiments performed upon the arteries of living animals, and by the result of surgical operations upon the human subject, and the dissections after death; and the injected preparations contained in our anatomical museums exhibit, for the principal arteries of the body, the place at which the main trunk has been rendered impervious, and the mode in which the circulation has been preserved.

In the chest, the aorta has been obliterated by disease, and the intercostal arteries have supplied its place in carrying on the blood. In the abdomen, the aorta has been entirely obstructed by an aneurism situated above the bifurcation; the two iliacs below being reduced to mere cords. The common iliac has been successfully tied by Mr. Guthrie; and the internal iliac by Mr. Stevens. The external iliac, and the arteries below it, have been now so frequently tied, and the anastomosing vessels so clearly demonstrated, that no doubt is entertained of an adequate supply of blood being sent to the lower limbs after these operations.

The subclavian arteries have but few anastomoses; but they are still sufficient to nourish the upper extremity; and the arteries of the arm below may be tied without danger of an insufficient supply by subsidiary currents.

The carotid arteries have been found, by Baillie, obliterated by disease. That artery has been now frequently tied on one side of the neck; and it has even been secured, at distinct periods, on both sides of the neck of the same person: and in these cases the current of the blood has still flowed freely into the remote branches.

Still, however, the intimate connexion between the functions immediately essential to life—of the brain and other organs, and the necessity for a due supply of blood for the maintenance of cerebral action, gives to the vessels of the head extreme importance in the eyes of the surgeon and physiologist, and justifies him in pushing his inquiries respecting them to the utmost limit.

It will be seen that some animals die immediately from interrupting the circulation in the carotid and vertebral arteries; but that others survive the experiment, and give an opportunity of ascertaining the means of anastomosis.

*Ligatures placed upon the vertebral and carotid arteries of a dog.* On the 28th of January, 1831, I tied the right and left vertebral and the right and left carotid arteries of a dog,. and all was completed within half an hour. The animal appeared insensible, or as if it were intoxicated; it had difficulty in breathing; its pupils were dilated; its volition was diminished; and it ran against the leg of the table, or any other body, without seeing or regarding it. When placed upon its legs, it fell down on its right side, and had spasmodic twitchings of its hinder extremities. At the expiration of a quarter of an hour, it was still insensible; it had shiverings, although placed near the fire; it rested its head upon the ground on the right side; its respiration was still laborious ; and its pupils were dilated. After an hour and a half, however, it was able to stand, and, although with difficulty, to stagger around a small room.

On the 29th, it was dull, and indisposed to move. On the 30th, it was much the same, and not inclined to move or eat. On the 31st, it walked round the room; and ate about an ounce of food, but would not lap. On the 1st of February, it was much better: it ate and drank; and from that time gradually recovered. It afterwards became a good house-dog; and I kept it for nine months, when it was killed, that I might inject it. The number and size of the anastomoses were very extraordinary. The description of them is as follows: The carotid artery on the right side was obliterated opposite the fifth and sixth carvical vertebræ: below the obliteration it is injected from the aorta; above the obliterated part it is filled with injection, (1) from the inferior thyroideal artery communicating with the superior thyroideal by large branches; (2) by a large descending cervical branch, dividing into numerous large anastomoses; and (3) by branches from the vertebral artery anastomosing with the external caratid artery on the first vertebra of the neck. The left carotid was obliterated from near its origin, but filled with injection above the obliterated part, by the inferior thyroideal artery communicating with the superior, and by the ascending cervical artery from the subclavian, by numerous and large anastomoses, and by an œsophageal artery from one of the intercostals communicating with the superior thyroideal artery. The right vertebral artery was obliterated near its origin on the seventh cervical vertebra, but filled with injection above the obliterated part by two branches from the superior intercostal arteries, which passed, on the back of the spine, into the arterial canal of the vertebræ, at the fourth, fifth, and sixth intercostal spaces. The vertebral artery thus produced passed to the second vertebra of the neck, where it formed the basillary artery, and, in its course, had festoons or loops formed in it, as far as the first vertebra, at each intervertebral substance; and here, upon the transverse process of the first cervical vertebra, it formed communications with the carotid. The left vertebral artery was obliterated close to its origin, but was filled with injection by an anastomosing branch from the superior intercostal artery, which entered between the fifth and sixth vertebræ of the neck; and by a second branch, also, from the intercostal artery passing on the posterior surface of the transverse processes of the fourth and fifth cervical vertebræ; then, over each transverse process was a loop of arterial communication, forming down each side a beautiful display of festoons.

On a second occasion, I tied the left vertebral artery of a dog. I then secured the right vertebral; and after an interval of eight days I put a ligature on each carotid artery. The animal was weakened in its forelegs; but in other respects it suffered less than the former; and on the following day it took its food as usual. The right carotid was obliterated; the injection passed from the aorta to the obstructed part, and above it, by an anastomosing vessel from the vertebral, and by an ascending cervical artery from the right subclavian. The left carotid was obliterated, but filled with injection to the place of obliteration, from the aorta; and above, it was filled by an ascending cervical, an inferior laryngeal branch, and others from the vertebral. The right vertebral artery was obliterated opposite the seventh cervical vertebra, before it entered the foramen of the sixth vertebra; but above the obliteration it was filled by an anastomosis with the superior intercostal artery: it then ascended through the canal in the sixth cervical vertebra, forming beautiful festoons and injunctions with arteries passing over the vertebra, opposite each intervertebral substance, and joining, by anastomosis, with the carotid at the first vertebra of the neck. The left vertebral artery was obliterated at the seventh vertebra; but the artery formed anastomosis, one with the subclavian, and two with the superior intercostal. The artery on this side formed similar but larger junctions than the right, opposite to each intervertebral substance, in festoons or loops; and thus the vertebral artery was reproduced, and filled with injection from these vessels. The two vertebral united to form the basillary artery as usual, and joined with the internal carotids at the circle of Willis.

Where the basillary artery was first formed, anastomoses were sent to the carotid arteries on the transverse process of the first cervical vertebra.

The result of tying the carotid and vertebral arteries in the dog is such as I have described; but in the rabbit it is different, as in this animal the arrest of the blood in these four vessels is immediately fatal.

Besides the determination of this point, it was my object, in the following experiments, to ascertain the different effects which would be produced by tying separately the vertebrals and the carotids. The size of the carotid arteries, compared with the vertebrals, is much less in some animals than in man, in proportion to the inferior developement of the cerebrum; and the tractus respiratorius being supplied by the vertebrals, the current of blood in these arteries might be supposed to exercise an influence on the respiration.

*Ligature placed on both carotid arteries.*—In the first place, I applied a ligature to the carotid artery on each side of the neck. Little effect was produced; except, that the respiration was quickened for a few minutes, and the animal rendered dull and disinclined to eat during the day; but on the following morning it appeared lively, and ran about with its natural activity; so that it may be truly said, that these two arteries may be tied with very little change in the functions of the animal, excepting that the respiration is quickened; and this perhaps may be attributed to a greater quantity of blood being impelled through the vertebral arteries, in consequence of its interruption in the carotids.

*Ligatures placed on both vertebral arteries.*—I next placed a ligature around both vertebral arteries. When I had tied the first, there was some difficulty in breathing; but when I had tightened the second ligature, this difficulty was greatly increased. The respiration was at first slow, but it afterwards became quicker. The animal retained volition and sensation, but its fore legs were weakened.

At the end of two hours, its breathing was laborious; its ears dropped to the right side; its heart beat quickly; it was dull, and indisposed to move; and its fore legs were still weak. After four hours and a half, it ran about, but with its ears fallen: its respiration was slower. On the following day, there was a murmuring in its breathing, which was increased under excitement: its heart beat quickly and forcibly: its pupils were not dilated. On the second day, the respiration was slow and heaving; it had an irregular action of the heart; it was dull; and its heat was 102. In the evening, its respiration was irregular and heaving; but it moved about, and took food. On the third day it was dull: its breathing was slow: its heart beat quickly: it ate food. On the fourth day it appeared heavy, and indisposed to move. The action of its heart was quick and strong; its respiration was slow, but no longer stertorous. On the fifth day, its breathing was slow; it appeared dull and heavy; the action of the heart was still quick. On the sixth day, the respiration was laborious and slow, being only 64, instead of between 120 and 150, the natural number of inspirations in a minute; its heart beat rapidly, and not forcibly; it was very dull, and indisposed to move; it was getting thin, but took its food as usual. On the seventh day the animal was found dead; and on the eighth I examined it, after injection, and found an abscess in the neck. The vertebrals had been well secured, and the brain had received injection by the carotid; the basillary and cerebellal arteries were filled from the circulus arteriosus. The animal's death may have been hastened by the abscess.

I have many times repeated this experiment; and it uniformly produces a marked effect upon the respiration, which it renders slow and laborious. The fore legs are weakened; and a much more severe effect is produced upon the animal than when the carotid arteries are obstructed; insomuch, that it will rarely recover from the operation.

*The carotids first tied, and then the vertebrals.*—The next step was, to ascertain the effect of arresting the blood in the vertebrals, after the carotids had been secured. I tied the carotid arteries; the respiration and circulation became quicker; volition and sensation remained in all their activity. In twenty-four hours, the animal appeared very lively, but its breathing was quicker than natural. After forty-eight hours it breathed less quickly; it ran about in a lively manner; and it ate heartily. On the third day it was difficult to catch; on the fourth, fifth, sixth, seventh, and eighth days, it appeared to be in a natural and healthy state; and on the ninth I exposed the vertebral arteries, and found them obviously enlarged. A ligature was tied upon each of these vessels. The respiration stopped immediately; and the animal appeared dead; but it afterwards made seven gasps, from convulsions of the diaphragm; its hinder extremities became also convulsed; but in a minute, all voluntary motion ceased.

On opening the abdomen and chest, it was seen that the peristaltic motion of the intestines remained; and the heart continued to act for a few minutes after apparent death.

This experiment shews how little the functions of the brain depend upon the carotids, and how much upon the vertebral arteries.

*Carotids tied—vertebrals compressed.*—As tying the vertebral arteries is a difficult experiment, it occurred to me that I might compress them with my fingers, after tying the carotids, and produce the same effects. I tied the carotid arteries. Respiration was somewhat quickened, and the heart's action increased; but no other effect was produced. In five minutes, the vertebral arteries were compressed by the thumbs, the trachea being completely excluded. Respiration almost directly stopped; convulsive struggles succeeded; the animal lost its consciousness, and appeared dead. The pressure was removed; and it recovered, with a convulsive inspiration. It laid upon its side, making violent convulsive efforts; breathed laboriously; and its heart beat rapidly. In two hours it had recovered; but its respiration was laborious. The vertebrals were compressed a second time. Respiration stopped; then succeeded convulsive struggles, loss of motion, and apparent death. When let loose, its natural functions returned, with a loud inspiration, and with breathing excessively laboured. In four hours it was moving about, and ate some greens. In five hours the vertebral arteries were compressed a third time, and with the same effect. In seven hours it was cleaning its face with its paws. In nine hours the vertebral arteries were compressed for the fourth time; and with the same effect upon its respiration. After thirteen hours it was lively. In twenty-four hours the vertebral arteries were compressed for a fifth time, and the result was the same, viz. suspended respiration, convulsions, loss of motion and consciousness. On the removal of pressure, violent and laborious respirations ensued; and, afterwards, the breathing became very quick. After forty-eight hours, for the sixth time, the compression was applied, with the same effect.

Thus it appears, that if the carotid arteries are tied, simple compression of the vertebrals succeeds in putting an entire stop to the functions of the brain.

*Vertebrals tied—carotids compressed.*—I then reversed the preceding experiment, by impeding the current of blood first in the vertebral arteries. I placed a ligature tightly around the vertebral arteries. The respiration became immediately laborious; its right ear fell, and the right fore leg was partially paralyzed. In one hour it was indisposed to move; its respiration was slow and laborious; and its right fore leg in a great degree recovered. Its sensation remained; but its volition was less than before the experiment; it smelt at the food offered, but would not eat it. In three hours green food was placed in its mouth, which it ate. In five hours it was running about; but its right ear remained in the same situation. On the following day, its respiration was slow, and it appeared dull. I pressed, with my thumbs, the carotid arteries on each side of the larynx, which was left free. It fell upon its side; it lost all sensation and volition; and its eyes were drawn back. Upon removing the pressure, it soon recovered. On the second day, its respiration was quick; its ear much risen; its fore leg less paralytic: it sat up; and moved from place to place. A second time I compressed its carotids. Its eyes were drawn back; it was convulsed; and its respiration was quick and laborious; and it was affected in the same way as on the preceding day, but in a less degree. On the third day its respiration was hurried, and 150. For the third time I compressed its carotids. It fell upon its side, and was insensible; but soon recovered, and ran about. On the fourth day it was dull, and its respiration was laborious; it ate some green food. In the afternoon of this day, it became very dull, and refused the food placed before it; and on the morning of the fifth it was found dead.

I injected this animal with coarse injection; and, upon dissection, it was found that abscesses had formed around the ligatures. The vertebral arteries were fairly tied, and the carotids greatly enlarged, but they were compressed by the abscesses. The injection had entered the cranium by the internal carotids, but not by the vertebrals; nor was there any injection in the basillary artery by the circle of Willis.

*Carotid and vertebral tied on the same side.*—On another occasion, I tied the carotid and vertebral arteries on the same side; the breathing became laborious, and the fore leg was partially paralyzed. I subsequently compressed the vessels on the other side, with the usual effect of producing apparent death: but the pressure being removed, the animal recovered; and at the expiration of eighteen days it was quite well, excepting that it had a difficulty in breathing under excitement. It was then killed and dissected. The arteries had been securely tied. It appears, then, that the obliteration of one carotid and one vertebral, on the same side, does not produce a fatal effect.

*Vertebral and carotid arteries tied at the same time.*—In order to cut off at once the several currents of blood to the brain, I tied at once both the vertebral and carotid arteries. The animal breathed no more; but there were thirteen or fourteen convulsive contractions of the diaphragm, and convulsions of the hinder extremities, and the animal ceased to exist.

This is a most decisive experiment; shewing the effect of the arrest of the blood in the vessels of the brain, in stopping respiration, volition, and sensation; and the result is striking and immediate.

The same effect of interrupting the streams in the vertebrals and carotids was produced in an equally conspicuous manner, without the application of ligatures, as follows. The animal was held in a convenient position, with its neck extended, and its head thrown back. I then applied my thumbs, so as to compress, at the same time, the two vessels on each side, taking care to leave the trachea entirely free from compression. Respiration ceased in a few seconds; some struggling then took place, and the animal appeared dead. The pressure being then removed, the respiration was completely suspended; but artificial motion being given to the ribs, the animal gasped, began to breathe quickly, and recovered.

I also put a ligature around the neck, close to the sternum, so as to compress the carotid and vertebral arteries; but the trachea was excluded, by passing the ligature behind it. Although the trachea remained free, as soon as the ligature compressed the carotid and vertebral arteries, breathing ceased, and all the functions of the brain were destroyed,

Before I would venture to draw conclusions from the experiments above detailed, I was desirous of convincing myself that no injury done to the nerves could have influenced, in any material degree, effects which had been observed. I proceeded, therefore, to investigate the consequences of applying ligatures to the principal nerves of the neck.

*Ligatures placed on the pneumogastric nerves.*—In the first place, I put a ligature on each pneumogastric nerve. The animal's breathing became heaving and laborious, and fell from 150 to 48 inspirations in the minute: it was likewise accompanied by a stertorous noise: the heart beat feebly, but rapidly; food was refused. These symptoms continued; and on the following morning it was found dead. The same experiment was several times repeated, and the results were nearly uniform, the animals dying at the end of from nineteen to twenty hours.

In these experiments, it was likewise observed that the blood circulating in the arteries gradually assumed the venous colour, and that the animal heat at the same time decreased in a remarkable degree.*

---

* These facts were carefully noticed in the following experiments.

EXPERIMENT 1, ON THE RABBIT. Respiration, 132 in a minute. Heat 104.

The pneumo-gastric nerve was tied on each side: the breathing became stertorous? the animal dull, and disinclined to move.

In 1 hour, respiration 48.
3 hours . . . . 44.
4 . . . . . . 56. Animal Heat 99¼ in the anus.
3 . . . . . . 48. . 93¼.

I opened the carotid artery, and blood of a venous colour escaped. I tied the artery.

The examinations after death exhibited the lungs gorged with blood, and looking like the liver; a fluid in the pleuritic cavity; the stomach full of undigested food; and the œsophagus likewise distended with it, in those cases in which the animals had taken food after the ligatures had been applied.

*Ligatures placed on the phrenic nerves.*—In another rabbit, I divided the phrenic nerves one after the other, in immediate succession.

The diaphragm being then paralyzed, the animal's respiration, which was performed by the intercostal muscles, instantly became excessively laborious. The ribs were heaved violently; and a much greater effect was produced on the respiration than when the vertebral arteries, the pneumo-gastric nerve, or grand-sympathetic, were tied.

In a quarter of an hour it lay upon its side, making great efforts with its intercostal muscles; and sometimes it stopped, as if fatigued, and then again commenced. In twenty minutes it was dead. On examination the phrenic nerves were found to be completely divided.

The heart's action, and the peristaltic motion of the bowels, were observed for a short period after apparent death.

In this experiment, respiration was rendered difficult, by obstruction to the mechanical apparatus destined to provide the necessary supply of fresh air; whereas, in the former, the difficulty arose from the failure of those processes which in health are carried on within the lungs; and may we not thence infer, that the changes of the blood are not chemical alterations merely, but dependent also upon the vital agency of the nerves and blood-vessels?

---

In 11 hours, respiration . . . . . . . . . 36. Animal Heat 93.
12⅓ . . . . . . . . . . . laborious, and 30. . . . . . . 9¼ to ½
12 . . respiration the same. . . . . . 89.
The rabbit died at this time, and the heat of the abdomen was 88½.

*Examination.* Lungs loaded with dark blood: water in each cavity of the pleura: food in the œsophagus: stomach loaded with food.

EXPERIMENT 2. The pneumo-gastrict nerves tied. Respiration 135 before the experiment, Animal Heat 102.

In 1 hour respiration 48. Animal Heat 99.
3 hours . . . 39. . . . . . 99.—(Stertor, or moaning, under excitement.)
4 . . . . . 33. . . . . . 98½.—(The animal dull, and disinclined to move.
6 . . . . . 36. . . . . . 96: cold to the touch,
In 12 hours respiration 36. Animal heat 97½.
14 . . . . . . . . 32. . . . . . 95½.
16 . . . . . . . . 28. . . . . . 93.
16¾ hours the animal died. It heat 87 in the abdomen at the time of its death.

The animal felt cold long before it died.

The gradual decrease of the animal heat, the dark blood circulating in the arteries, and the gorged state of the lungs after the application of a ligature to the par vagum, are interesting and important circumstances: and we are led to question, whether the lungs, by this operation, are deprived of a nervous or vital influence essential to the change of the blood; or whether this change is not produced in consequence of the slowness of the animal's breathing; for when the phrenic nerves have been tied, the blood also becomes dark in the arteries. In that case, however, the lungs are not found gorged by blood.

*Ligatures placed on the grand-sympathetic nerves.*—I now tied the grand-sympathetic nerve on each side. The respiration became quick and irregular; but sensation and volition were unaffected. The heart's action was very quick: there was a general trembling; but when the animal was put down upon the ground, it ate some greens. Its respiration continued irregular, and its heart's action very quick; and after eight days it was killed. The nerves were found well tied: one had already ulcerated below the ligature; the other was nearly ulcerated through; and the ligature was surrounded by suppuration.

In another rabbit, I tied these nerves; and the animal, although it is near a month ago, is still lively and active.

*Ligatures placed on the pneumo-gastric, phrenic, and grand sympathetic nerves.*—Lastly, I tied, in one rabbit, the pneumo-gastric nerve, the grand-sympathetic, and the phrenic. The respiration became laborious; the animal dull, and indisposed to move; and the heart's action feeble. The breathing continued excessively laborious for a quarter of an hour, when the animal died. Bloody fluid was found in the chest: the lungs were not much changed. In another similar experiment, the animal died in three quarters of an hour.

We see, then, that an animal with all these nerves compressed may live from a quarter to three quarters of an hour; that the ligatures on the pneumo-gastric kill in about twelve hours; and in the grand-sympathetic, that the animal will continue to live for a much longer period: so that pressure on these nerves, in the experiments in which the arteries were compressed by my thumbs, could not have been the cause of death.

The effect of tying the jugular veins of the rabbit is not constantly the same in all cases, as the following instances prove.

*Ligatures placed on the jugular veins.*—In one rabbit, I tied the jugular veins on each side of the neck. When it was set at liberty, it ran about, cleaned its face with its paws, and took green food.

Its respiration was reduced to 68 inspirations in a minute, which is about half the natural number. After four hours, it ran about as if nothing had happened, and eventually recovered.

When it was killed and injected, I found, on each side, three anastomosing veins passing from the anterior to the posterior part of the jugular vein, and conveying the blood from the head to the heart; but the vertebral vein had remained whole, and become enlarged; and it passed on the fore part of the vertebræ, from the head to the space between the fourth and fifth cervical vertebræ, where it entered the vertebral canal.

In a second rabbit, I tied the jugular veins on each side of the neck, as before. The animal's respiration became slow; but it ate green food, ran about, and was difficult to catch; but for five days after, it appeared dull; its ears had dropped. On the seventh day, it was seen to be convulsed, and frequently rolled over. Its voluntary powers were lost, as well as its sensation, in a great degree. On this day it died. On examination, a clot of blood was fonud extravasated in the left ventricle of the brain.

Hence it follows, that apoplexy will occasionally result from an obstruction to the return of blood in the jugular veins; and this I have known to happen from enlargement of the glands in the neck of a boy.

*Inferences drawn from the foregoing experiments.*—It appears, from these experiments, that the carotid arteries are designed in these animals rather for the supply of blood to the external parts of the head than to the brain itself; whilst in proportion as the brain is more developed, the carotid artery acquires greater importance. The obstruction of it influences respiration in some degree; probably, because, under these circumstances, more blood is directed to the vertebral arteries. The internal carotid branches are proportionably less than in those animals which have a large cerebrum, and are endued with higher mental faculties.

It passes to its destination, to prevent the action of the heart from influencing the brain immediately.

The rabbit quickly recovers from the operation of tying these arteries, and seems little affected by it: and in man, as well as in these animals, these vessels are obliterated without the destruction of life.

The vertebral arteries are much more important vessels, as regards the brain and its functions in these animals,* than the carotid arteries. The nervous power is much lessened by tying them; and, in these experiments, the animal did not, in any case, survive the operation more than a fortnight; although I do not mean to say that recovery is impossible. In the dog, also, the carotids may be tied with little effect; but the vertebrals have a great influence.

The effect of the operation is, immediately to render breathing difficult and laborious, from the supply of blood to the phrenic nerves and the whole course of the tractus respiratorius of Sir Charles Bell being stopped. The animal becomes dull, and indisposed to use exertion or to take food.

Very slight injuries, after a ligature has been put upon these arteries, will destroy life; insomuch, that if they are first tied, even dissecting for the carotids, without tying them, will cause death. The best method, in such experiments, is to tie the vertebrals last.

On account of the importance of these vessels, they are securely defended by bone in the greater part of their course; and it is only below the sixth cervical vertebra that they are accessible. If they were exposed to pressure, death would often be suddenly produced.

These arteries are tortuous, to prevent too sudden a rush of blood to the head; and they pass through foramina of bone, which prevents any great increase of their size; although they become somewhat larger than before, when the carotids are tied, and *vice versa.*

Thirdly, compression of the carotid and of the vertebral arteries at the same time in the rabbit, destroys the nervous functions immediately. This is effected by the application of the thumbs to both sides of the neck, the trachea remaining quite free from the pressure; when respiration entirely ceases, with the exception of a few convulsive gasps.

The same fact is evinced in a clearer and more satisfactory manner,

* Mr. Coleman informs me that the vessels of the horse are different; and he thinks it is designed to counteract gravitation, when the animal is feeding on grass.

by the application of ligatures on the four vessels, all being tightened at the same instant; when stoppage of respiration and death immediately occur.

When the dog is the subject of this experiment it loses its volition and sensation, and appears as if it were intoxicated; but the anastomosing vessels gradually restore the circulation by means of the other branches of the subclavian artery at the back and sides of the neck.

But, notwithstanding the decisive nature of the last experiment, conceiving that it might be possible that the pressure upon the nerves of the neck might have an influence in killing the animal suddenly, I made the experiments which I have detailed.

I first tied the pneumo-gastric nerves, and found that the animal lived about twelve hours, although it died on the instant when the carotid and vertebral arteries were tied: the lungs were also loaded with blood,* and twice as heavy as the healthy lung: it appears, therefore, that the change of the blood is either directly or indirectly under the influence of the par vagum.

In this experiment it is also to be observed, as a point of much importance, that the blood in the carotid arteries is found of a venous character, and dark blood circulates in the animal for some time before it dies, the blood being less arterial as the time elapses from the application of the ligature: yet the heart continues to beat; for when the artery is opened, the blood flows *per saltum.*

The blood also flows of a dark colour when the carotid is opened after the phrenic has been tied; but the lungs are not in that case found loaded with blood, but possessing their ordinary weight and appearance.

In this experiment there was also a remarkable diminution of animal heat. Is this to be attributed to a cessation of that pulmonary process, accompanied by the evolution of heat wherein venous is converted into arterial blood? or does it arise from a want of that supply of arterial blood to the nerves from which they derive a capability of evolving caloric? or shall we not approach still nearer to the truth, in supposing that both these causes of a high temperature are suspended at the same time; and that there is, consequently, a double reason for the gradual departure of the animal heat observed in this experiment?

The œsophagus contained food, in some instances in which the animal had eaten after the experiment, from its muscles being paralyzed; and the stomach was full, from the arrest of the digestive function.

This nerve, then, is most important; 1st, in assisting in the support of the function of the lungs, by contributing to the changing of the venous into arterial blood: 2dly, in being necessary to the act of swallowing: 3dly, in being very essential to the digestive process.†

The pair of nerves upon which I next applied ligatures were the phrenics. As soon as these were tied, the most determined asthma was produced; breathing proceeded by means of the intercostal muscles; and the chest was elevated to the utmost by them; and in expiration,

---

* Sir Benjamin Brodie has mentioned this state of the lungs.
† A ligature on one nerve only does not destroy.

the chest was as remarkably drawn in. The animals did not live an hour: but they did not die suddenly, as they do from pressure on the carotid and vertebral arteries. The lungs appeared healthy; but the chest contained more than its natural exhalation.

When the grand sympathetic was tied, little effect was produced: the animal's heart appeared to beat more quickly and feebly than usual; but of this circumstance I cannot be positively certain, on account of the natural quickness of its action. The animal was kept seven days; and one nerve was ulcerated through, and the other nearly so, at the situation of the ligatures. The suppurative process was extensively set up around the ligatures. No particular alteration of any organ was observed on examination. Another animal still lives in which the sympathetic was tied nearly a month ago.

Lastly, I tied all three nerves on each side the pneumo-gastric, phrenic, and grand-sympathetic; and the animal lived little more than a quarter of an hour, and died of dyspnœa.

The sudden death, then, that takes place from pressure at the sides of the neck must not be attributed to an injury to the nerves, but it is owing to the impediment to the due supply of blood to the grand centre of nervous influence.—*Guy's Hospital Reports*. No. III. *Am. Jour.*

---

2. *Physiological action of Iodine and Hydriodic Acid.* By ANDREW BUCHANAN, M. D.—The effects of iodine on the animal economy have not been discriminated with sufficient care from those of hydriodic acid, although the two substances, considered as physiological agents, are just as distinct as chlorine and muriatic acid.

The physiological effects of iodine are exceedingly similar to those of chlorine. It acts as a corrosive irritant, exciting inflammation and combining chemically with the tissues to which it is applied. This simple local action is all that, strictly speaktng, can be ascribed to iodine, for there is not the least reason to suppose that it is ever absorbed and mingled with the circulating fluids in the uncombined state. The other effects which have been ascribed to iodine are either those of hydriodic acid, or they are secondary effects arising from the inflamed or ulcerated state of the alimentary canal,—which the iodine has produced. To the latter series of effects must be referred the whole of the fearful train of symptoms which have been described by toxicologists under the name of *iodism*, and have been attributed, but most erroneously, to the slow accumulation of the poison in the body. This conclusion appears to me to be fully warranted by considering, that while such symptoms have been observed to result from the use of iodine in forms capable of exciting local irritation, it has been ascertained by numerous trials made in the hospital and out of it, as well by myself as by various medical friends who have done me the favor to state the result of their observations, that no such symptoms result from the use of iodine divested by starch of its local irritant power. Many of the symptoms, too, which have been described, such as indigestion, pains of the stomach and bowels, emaciation, and febrile excitement, are of a kind very likely to result from the gastro-intestinal irritation; and with respect to the rest of them it is difficult not to entertain a suspicion that some of them at least may have been admitted as effects of iodine from inaccurate observations. However that may be, I can only say, that I have never seen the use of iodine followed by wasting of the testicles or of the mammæ, by palpitations, faintness, excessive debility, hurried, anxious breathing, dinginess of the skin, copious clammy sweats, increased menstrual discharge, or an oily appearance of the urine, which are enumerated among the symptoms characterizing the supposed affection termed iodism. Some of the symptoms described, indeed—such as bilious diarrhœa, and a diminished secretion of the saliva, are the very reverse of the effects which I have usually observed to flow from the use of the preparations of iodine.

I go on to speak of the effects of hydriodic acid, which I think it does not admit of doubt ought to be regarded as the true medicinal agent, possessing the alterant virtues which have been ascribed to iodine itself. The hydriodic acid, in the concentrated state, is most probably, like the muriatic, a corrosive irritant, but by dilution with water it is completely divested of its irritant quality. In the diluted state, however, it is probably not without a local action on the alimentary canal. Most probably it acts as a tonic. At all events the analogy of the other mineral acids, in which tonic virtues are so generally recognized, warrants the opinion, as at least probable, that it is to the hydriodic acid that ought to be referred the tonic effects which so many observers have described as resulting from the use of iodine.

The hydriodic acid, whether prepared within the body, or introduced ready made, is an exceedingly absorbable substance. When given in doses equivalent to ʒij. of iodine daily, the whole of it appeared to be taken up and mingled with the circulating fluids. What are the fluids with which it mingles, and the secretions by which it is discharged? I have already mentioned, that when hydriodate of potass is given in a dose of ʒij., iodine is found abundantly in the blood in from four to six hours afterwards. I must also, however, mention, that when iodine is given in smaller doses, although the use of it be continued for a great length of time, no iodine can be detected in the blood on the most careful examination. Mr. Lumsden, to whose kindness and analytical skill I have to acknowledge my obligations, examined both the serum and crassamentum in several patients taking iodine in full doses, and in many of whose secretions it was contained in abundance, without being able to detect any trace of it, How to explain these facts I do not know, unless we are to suppose that iodine does not find its way into the sanguiferous system unless when given in such a quantity at once that the system is completely deluged with it; but that when given in smaller quantities it is not admitted by the extremities of the veins, and is transmitted along the capillary vessels in a way which we do not at present understand. It will be seen immediately that the vessels of certain excretories appear to possess a similar power of rejection over this substance. I have already mentioned, that after giving a full dose of the iodide of potassium, iodine was detected in abundance in the serum exhaled into the serous cavities, and in the synovia of the knee-joint. Of the secretions, it was in the urine that iodine was always detected in greatest abundance. It appeared in the urine about four hours after the first dose was taken, and continued to be detected in it for four days after the last dose was taken. In one or two instances it was found on the fifth and even on the sixth day after. It continued to be observed for the same length of time whether the iodine was given in one large dose, or the body had been gradually impregnated with it. Next to the urine it was in the saliva that the iodine was most abundantly found. It was found also invariably in the tears, and in the mucus of the nose, although in the latter case it was impossible to determine whether it was really secreted by the schneiderian membrane, or came down from the lachrymal gland. Iodine was found also in the milk, but it existed in that secretion in a very small proportion compared with that in which it was found to exist simultaneously in the urine and saliva. Iodine was also found in examining the mucus secreted from the lungs in chronic bronchitis, but it was difficult to determine whether its presence was not owing to an admixture of saliva.

While the hydriodic acid is thus widely diffused over the body, it would be to generalize too hastily to infer that it exists in all the animal fluids. I have already mentioned, that it is only in certain circumstances that it is to be found in the blood, and there are certain secretions the vessels preparing which appear constantly to reject it. At all events in none of the trials made could any portion of iodine be detected in them. Of these secretions or exhalations the first to be mentioned is the perspiration. I am aware that iodine is said to have been found in the perspiration, but I can only say, thst in experiments carefully made and frequently repeated upon patients taking iodine in very large doses, and whose urine and saliva became as black as ink on being tested in the usual way, not the slightest trace of iodine could be detected in the perspiration. The perspiratory fluid examined in these experiments was forced out by the use of diaphoretics, or was collected from the forehead of the patient in the hot bath. At the same time the water of the bath itself in which the patient was immersed, was examined, but with no better success. It may, therefore, I think, be confidently affirmed, that iodine is very rarely, if ever, present in the perspiration. Another fluid, in which I very early looked for iodine, and fully expected to find it, was the purulent matter secreted from sores, for the cure of which iodine was exhibited, but in none of very numerous experiments made on patients fully saturated with iodine, could the slightest tinge of the medicine ever be perceived in the purulent discharges. The absence of iodine from the discharges from the skin, and from ulcerated surfaces, is rendered the more remarkable by its efficacy in the cure of cutaneous diseases, and various forms of ulceration. I have only to add further, that in one or two experiments, iodine was not found present in the serous fluid of blisters.

The whole of the hydriodic acid introduced into the body is discharged along with the urine, with the exception of the very small proportion of it contained in the rejected saliva and mucus of the nose, and milk, when that secretion is going on in the female. The greater part of the saliva being swallowed, the iodine contained in it must be absorbed a second time from the stomach and bowels. A similar re-absorption must take place of the iodine contained in the exhalations that are re-absorbed by the surfaces which effuse them. Thus, after describing many circuits, in various directions, through the body, nearly the whole iodine introduced comes ultimately to be discharged with the urine. The time occupied in this circuitous passage of the iodine is, as has been already remarked, generally four days.

Iodide of potassium is absorbed very readily from the skin: it is found in the urine just as when it is introduced into the stomach, but much less speedily.

With respect to the influence of the preparations of iodine we are here considering over the organs of digestion and assimilation, that influence was, to say the least of it, certainly not of an unpropitious kind; on the contrary, in patients employing these medicines in full doses, the tongue was almost invariably free from fur, and of a healthy red color; the appetite and digestion were good, and in many instances there was a most obvious improvement in *condition*. Of the two great alterant medicines we possess, iodine and mercury, it is certainly a most important advantage of the former over the latter, that it admits of being given freely, not only without injury, but with advantage to the general health; while mercury, given in full doses, is always a dangerous medicine, and often the means of doing irreparable injury to the constitution. The iodide of starch frequently caused costiveness, attended with griping pains of the bowels; and, as was observed in many instances, with a paleness, approaching to a clay color, of the alvine discharges.

These effects I was inclined to refer to the large quantity of hydriodic acid generating in the stomach, abstracting the free soda from the bile, and probably otherwise modifying its qualities, besides, perhaps, exerting a direct astringent action on the bowels themselves. The occurrence of these symptoms required the use of a laxative. In some cases, but very rarely, the iodide of starch produced the opposite effect of causing purging. In persons of weak digestion, it was often necessary either to give up the medicine altogether, or at least to diminish the dose of it, owing to the occurrence of pain in the stomach. It is possible that the conversion of iodine into hydriodic acid requires a certain vigor of digestion; and if so, the pain occurring in such cases may have been owing to the imperfect mode in which the process of conversion was carried on.

A question has of late been agitated—Whether iodine ever causes salivation? It does not, according to my observations, generally do so; but that it does so occasionally, I think there can be no doubt. In a man who had taken 2864 grains of iodine in the course of 42 days, in the form of iodide of starch, the medicine required to be given up on account of salivation, which was as profuse as I ever saw caused by mercury, and attended with swelling of the face and ulceration of the inner membrane of the mouth. It only differed from a mercurial salivation in being much less obstinate, going off as soon as the iodine disappeared from the saliva. I never saw this symptom occur to the same extent in any other case, but I have repeatedly seen it in a less degree. The man in whom it took place had been frequently salivated with mercury on previous occasions.

In several cases the pulse was accelerated when the system was impregnated with iodine; in many other cases no acceleration could be perceived, Upon the whole, one of the most remarkable circumstances attending the use of this medicine was the absence of symptoms produced by it. Patients who took the largest doses went about, and took their food, just as usual: so that had it not been for the peculiar chemical condition of their secretions, and in many instances the rapid disappearance of their diseases, it would not have been supposed they were taking medicine at all.

With respect to the relative efficacy as medicines of the iodide of starch, hydriodic acid, and iodide of potassium, I am inclined to place them in the order in which they have just been enumerated; although I must admit that the superiority whichI ascribe to the first is perhaps owing to my having prescribed it most frequently. The action of all of them, however, is very similar. The only mode of explaining the similarity of action on the body of substances so dissimilar in nature, is by considering the hydriodic acid as the active principle, producing the physioiogical and therapeutical effects usually ascribed to iodine. It has been already stated, that free iodine is immediately converted in the stomach into hydriodic acid. As to the hydriodate of potass, it is clear that in its passage through the body the muriate and phosphoric acids must lay hold of the potass, so that the hydriodic acid will combine with the weaker bases, the soda and lime, and thus pervade the system nearly in the same state as when free hydriodic acid itself is employed. The presence of the potass, however, must produce an important difference in the action of the hydriodate, which I have no doubt a more attentive observation will hereafter enable us to discriminate.

If the hydriodic acid be the active principle to which the alterant virtues ascribed to the preparations of iodine are to be attributed, analogy will lead us to range those preparations in the series of medicinal agents along with the muriatic, the sulphuric, the nitric, and other mineral acids,—all of which have been held to possess alterant virtues, and have been used on that account in syphilis and other diseases; and the great success which has attended the use of the hydriodic acid may serve as an inducement to try the other mineral acids more extensively, and in fuller doses, than they are commonly prescribed.—*Med. Gaz.* July 2, 1836. *Ibid.*

---

3. *Iodide of Potassium.*—Dr. Buchanan says he has given this substance in doses of half an ounce, and the only precaution he observed was to máke the patient drink freely of diluents. "No pain of the stomach or bowels was," says he, "produced, and the medicine never in the least degree operated as a purgative, but seemed to be altogether absorbed, and was discharged chiefly by the kidneys."

As the iodide of potassium is very often adulterated, it is necessary, to prevent any suspicions from arising in the mind of the reader as to the purity of the medicine employed, to state, that the iodide of potassium used in the Glasgow Infirmary is prepared under the direction of a most able chemist, within the walls of the hospital, where there can be no motive to adulterate it.

It was not merely for the purpose of determining the dose of the hydriodate of potass that these large quantities of it were given. I found that to give it in such doses was the readiest mode of determining certain physiological questions with respect to the diffusion of this medicine over the body; for so large a quantity of it being at once introduced into the system, every fluid into which it is permitted to enter, is at once impregnated with it. It is in this way that it is most easily detected in the blood. Two drachms of it were given to a young man affected with gonorrhœa, and as soon as the medicine made its appearance in the urine, which was four hours afterwards, blood was drawn from his arm. On examining the blood, both the serum and crassamentum were found deeply impregnated with iodine. The same dose was given to a boy affected with dropsy of the knee-joint, from which it had been resolved to draw off the fluid. About five hours after the dose had been taken, a very small puncture was made into the joint, and upwards of twelve ounces of synovia drawn off by the cupping-glass. The synovia contained iodine in abundance. To an old man who had one of the largest hydroceles I ever saw, two drachms of the hydriodate of potass were given over night, and the same quantity the following morning. On tapping him some hours after he had taken the last dose, fully more than thirty ounces of serum were discharged, containing a large quantity of iodine.—*Ibid. Am. Jour.*

---

4. *Results of experiments with Kreosote.*—Dr. J. Corneliani, Professor of Internal clinic in the University of Pavia, has investigated the powers of the kreosote experimentally, both in his clinic and on several species of animals. He has employed it internally, endermically, and finally by injection into the veins; and has varied the dose from the smallest to the highest. The following are his results:

1. Kreosote taken internally, in a large dose, may produce instantaneous death, without any organic lesion being observed on post mortem examination, if made immediately.

2. When pure or very slightly diluted kreosote is applied directly to a larger nerve, as the par vaguum, or it is injected into a vein even in small quantity, death instantly follows.

3. If the quantity is not sufficient to produce death, it causes prostration of the muscular and nervous systems; and symptoms of paralysis of the extremities, heart, diaphragm and organs of the senses, which leads to the belief that the kreosote acts like the narcotic debilitants, among which it should be arranged.

4, Although no antidote has yet been indicated against its toxicological effects, it appears that general stimulants are indicated, unless it has acted upon the stomach; in that case the debilitants, and especially narcotics, as the prussic acid, increase its fatal effects.

5. The kreosote also exerts more or less active mechanico-chemical action on the gastro-enteritic mucous membrane, which gives rise to various organic lesions met with in the dead body, and to chronic gastro-enteritis in which persons are subject who make use of this article for a long period.

6. To counteract this mechanico-chemical effect produced by the kreosote taken internally, oleaginous and mucilaginous drinks should be administered. Vinegar being an excellent solvent of this substance—prevents its deleterious effects by favoring its contact with the nervous papillæ of the stomach.

7. In general, patients cannot bear a larger dose than two drops, repeated four or six times in the 24 hours.

8. Kreosote taken internally, may be useful in diabetes mellitus, polydipsia, hemoptysis, chronic catarrh, diarrhœa, palpitations of the heart, inflammatory fevers *(angiotenies)* and perhaps also in tetanus.

9. A singular effect of the kreosote taken internally appears to be its prompt action on the urinary organs; for the animal experimented on, urinated as soon as he took it. Dr. Corneliani ascribes this to the paralysis it causes, especially of the neck of the bladder.

10. The application of kreosote to the exterior in chronic inflammations of the skin, and especially in herpes (dartres), itch, chronic psoriasis, may be often useful, whether its action is drying, antiphlogistic or insecticide.

11. If the kreosote used externally is not applied to a large surface or upon important nervous branches, it produces no alteration of the organs, even of those upon which it more especially acts, as the spinal marrow, the brain and the kidneys. Dr. Corneliani has however observed that in phthisical patients, the inspiration of the kreosote may give rise to prostration of the intellectual functions and muscular movements.

12. If the external employment of the kreosote does not arrest venous hemorrhage, at least that of a large vein, as the femoral of a sheep, it is efficacious in hemorrhage from an artery of moderate size.—*Journ. de Chimie Medicale*, Feb. 1836. *Ibid.*

—

5. *Iodide of Starch.*—Dr. Andrew Buchanan, Junior Surgeon to the Glasgow Royal Infirmary, in a communication in the *London Medical Gazette* (2 July, 1836) extols this preparation of iodine, which he prepares in the following manner:—℞ Iodine gr. xxiv.; Amyli in pulveren tenuissimum triti ℥j. The iodine is first triturated into a little water, and the starch gradually added, the trituration being continued till the compound assumes a uniform blue color. The iodide is then dried with a heat so gentle as not to drive off the iodine, and it must be afterwards kept in a well stopped bottle.

The iodine, in the usual forms of exhibition, cannot in general be safely given in larger doses than four or six grains daily; whilst in the above formula Dr. Buchanan has given as much as 72 grains daily. Dr. Buchanan is in the habit, in persons not laboring under dyspepsia or constitutional delicacy of habit, and whom he wished to put under the influence of iodine, of commencing with the iodide of starch in doses of half an ounce three times a day, and increasing it immediately afterwards to ounce doses if necessary. "As there is no great reason," he observes, "for nicely apportioning the doses, a heaped tea-spoonful, desert-spoonful, or table-spoonful are convenient enough measures for smaller quantities in private practice. I have always directed the medicine to be taken in a draught of water gruel."

It might rationally enough be supposed from the large doses in which the medicine is administered by Dr. Buchanan, that it is an inert preparation, but such seems not to be the case, as Dr. B. has detected the iodine abundantly in the secretions, showing it to be absorbed; and has been unable to detect any traces of it in the stools of patients taking it in large doses. It certainly must exert a less irritant action on the alimentary canal than the other preparations of iodine, but it may be a question whether it will be possible to induce patients to persevere in a medicine requiring to be given in such large doses, making a meal rather than a medicine! *Ibid.*

---

6. *On Dropsy following Scarlet Fever.*—By J. Stark, M. D., Edinburgh. Dr. Stark has given an excellent account of the scarlet fever as it prevailed epidemically in Edinburgh, in the autumn and winter of 1835–6. In fifteen cases dropsical sequelæ followed. In the most severe cases, the swelling came on suddenly without any previous complaint, and from a fortnight to three weeks after the disappearance of the eruption. The patient went to bed well, and in the night or the next morning the friends were alarmed by the sudden swelling of the body, attended with dyspnœa, moaning, restlessness, and sometimes stupor. In all the cases in which the urine was examined, it was found coagulable by heat. In many of the severer cases, the urine passed before the dropsy came on was very turbid, dark colored, in a few cases bloody. In two cases it was suppressed. Exposure to cold seemed the immediate cause of the dropsy: the function of the skin being checked, the blood is thrown upon the internal organs, and the kidney, whose action is vicarious with the skin, particularly suffers.

In all cases, except the very mildest, he bled in proportion to the severity of the symptoms, and the relief experienced from it. In severe cases he bled from the arm until the pulse was affected: under two years of age, leeches were used. The bleeding was followed by small doses of the antimonial wine with the liquor ammon. acet., and, where the urine was suppressed, a large hot bran poultice was applied to the lumbar region. In milder cases, the same medicines with brisk purging. The warm bath was of the most advantage. There was but one fatal case, of a boy five and a half years old, whose parents would not permit bleeding or leeches. Dissection was not allowed.

[The explanation of the action of cold in producing dropsy is given by Dr. Stark as his own, on which he founded his practice, which was very successful. It is but justice, however, io Dr, Osborne to state that he had previously advanced an identical explanation. (See British and Foreign Medical Review, vol. ii. p. 222.)]—*Edinburgh Medical and Surgical Journal,* October, 1836. *Bell's Jour.*

---

7. *Efficacy of Chlorine in Scarlatina,—To the Editor of the Medical Gazette.*—Sir, Owing to the constant prevalence of scarlet fever, I am induced again to call the attention of the profession to the employment of chlorine in this too often fatal disease. A few years ago, when I resided at Bromley, in Kent, I published in the Medical Gazette some very remarkable cases of scarlatina, treated by my partner, Mr. Williams, and myself, in which the chlorine appeared to be highly useful. Not having the Gazette by me, I cannot recollect the exact time, but I think it was in September, 1830. However, I will venture to trouble you again with the particulars of one very striking case. It was that of a young woman in the service of Lord Farnborough. She had been to visit her parents in the country. Whilst there, her brother and sister died of malignant scarlatina. Soon after her return to Bromley Hill she fell ill, and had the disease in its worst form. I visited her first on a Sunday evening: I gave her an emetic of ipecacuanha, and then directed her to take a glassful of the chlorine mixture as frequently as she could swallow it. Under this treatment the dangerous symptoms soon gave way, and on the Saturday following she was down stairs.

I could relate many more cases, in which equal success attended the use of this medicine. In short, out of a great number of patients who were at that time attacked by the disease, two only died, both in the village of Wickham, one an infant, the other a spoiled child, ten years old, whose parents would not allow either medicine or nourishment to be given against the child's inclination.

I hope I shall be excused for again expressing a wish that medical men will give this medicine a fair trial, as other means so often prove ineffectual. My practice is to give, in the first place, an emetic of ipecacuanha, according to the age of the patient; to avoid active purgatives, which are injurious to children (particularly large doses of calomel;) and then to give the chlorine as frequently as possible.

As it appears to me highly important that the chlorine should be well prepared, I will subjoin the formula for that which I use, which was given to me by Mr. Brown, surgeon, at Lewisham:—

℞. Potass. Oxymur. ʒij.; Acid. Muriat., Aquæ Distillat., aa. ℥ij.

The acid and water to be mixed, and the oxymuriate dissolved in them. It is to be kept in a stopper bottle, in a dark place. Two drachms of this solution are to be put into half a pint of distilled water, to make the chlorine mixture.

I remain, sir, your obedient servant, R. T. Taynton.

39 *Queen Square*, Jan. 25, 1837. *Ibid.*

---

8. *New Method of reducing Luxations of the Humerus.*—By M. C. Gerard. (Thesis.) M. Gerard has employed the following plan to thirteen cases of dislocated humerus, during the last fifteen years. All the cases were recent; he thinks it advisable in all the kinds of dislocation. The patient being seated in a chair, an assistant, placed on the side opposite to the luxation, passes his arms around the neck of the patient, and, crossing his hands over the luxated shoulder, opposes the efforts made by the surgeon to replace the bone. The surgeon, stationed on the injured side, places his left forearm beneath the upper part of the dislocated bone, as near as possible to the armpit. He then approaches his patient so closely as to allow the cubital end of the dislocated humerus to rest against his own side, whilst he supports it longitudinally as near as possible to the trunk of the patient. The surgeon then draws the articulation in a direction upwards and outwards, and, without using much force, the bone is immediately replaced.—*Archives Gen, de Med.* Juillet, 1836. *Ibid.*

---

9. *New Treatment of Strictures of the Urethra.* By M. Jobert, Surgeon to l'Hospital St. Louis.—Having ascertained the situation of the stricture, M. Jobert oils a bougie, and then dips it into calcined alum reduced to an impalpable powder: if the obstacle is considerable, he dips the bougie again in oil, and afterwards in calcined alum, so that there are two layers of alum upon the bougie. He introduces the instrument gradually, and presses is softly against the obstacle, and fixes it in the urethra by four tapes. Sometimes two hours are sufficient to conquer the obstruction, and allow the patient to pass his water; at other times, it does not succeed until the next morning. It is necessary to introduce the bougie, similarly medicated, for many days successively, until it reaches the bladder. M. Jobert has found the most obstinate strictures yield to this treatment: the inflammation it produced was moderate, and the discharge soon ceased.—*Journal Hebdomadaire des Sc. Medicales*, 10th Sept. 1836. *Ibid.*

---

10. *New Treatment of Onychia Maligna.* By C. Ray, Esq., Falkingham.—Mr. Ray is opposed to the entire revulsion of the nail in this most troublesome disease, and justly states that it is useless to expect a cure until that part of the nail, at least, which acts as a constant source of irritation is removed. His mode of treatment is, after freely, dividing the swollen parts over the root of the nail and cauterizing them to apply poultices, and enjoin perfect quiescence of the limb. This mode of treatment, the caustic being re-applied every second or third day, brings the part into a state favorable for the partial removal of the nail, which is effected by passing a scissors from within outwards beneath the posterior angles on each side, and removing "an exact triangular portion," the two incisions meeting in a point in the centre of the posterior edge of the nail. After the separation of the nail, the wound is dressed with adhesive straps which are not to be removed for some days.—*Lancet*, November 12, 1836. *Ibid.*

---

11. *On Binding the Abdomen of Lying-in Women.* By Hugh Ley, M. D.—Dr. Ley is of opinion that the bandage so commonly applied after delivery has been greatly over-rated. In his experience there is but one circumstance in which the bandage with a compress over the uterus is imperatively called for. It is in a form of hemorrhage described by Dr. Gooch, in which the uterus which had contracted is again dilated by hemorrhage taking place into its cavity: in these cases, it is not only requisite that the uterus should be cleared of the coagula, but that it should be prevented from again enlarging; and this can only be done by a bandage and compress; but they should not be applied until repeated gushes of blood have reduced the circulation. In preventing the occurrence of hemorrhage, for which it has been advised, its use is very equivocal; for, amongst the poor where it is omitted, the relative mortality from hemorrhage is probably less than among the rich. To restrain actual hemorrhage it is obviously insufficient; and, if it may have been applied, it must be removed, in order to ascertain the state of the uterus, and to adopt those manipulations by which the bleeding is effectually restrained.

The bandage alone may be applied over the abdomen, with moderate and equal pressure, in cases of syncope following rapid delivery, which is produced, as in tapping, from the sudden removal of pressure; and, in such circumstances, it should be applied immediately on the expulsion of the child. It may also be used to satisfy ladies who believe that it will preserve their form by preventing permanent distention of the abdomen, and to relieve uneasy sensations consequent on the relaxed condition of the abdominal muscles: but, in these cases, it need not be applied until the clothes have been changed, and the patient placed in bed, and sufficiently loose to allow the hand to pass easily between it and the belly.

The bandage and compresses often produce *injurious consequences.* Dr. Ley has known it force the uterns to one side of the abdomen, producing much uneasiness in the hip, stretching the ligaments and pressing the appendages of the uterus against the sharp linea ileo-pectinea: hence laying the foundation of permanent obliquity of the uterus, not unfrequently a cause of barrenness. Another effect is to push the uterus lower into the cavity of the pelvis; thus producing prolapsus uteri, which Dr. Ley has more than once traced to this cause. Not only is the uterus pushed downwards, but the vagina is sometimes thrown into folds; the whole circumference descending until it forms a bulky tumor at the vulva, These evils are great, and not unfrequent. Dr. Ley is also inclined to think that the bandage increases after-pains.—*Med. Gazette*, September 10 and 17, 1836. *Ibid.*

---

12. *Nitrate of Silver in Diseased Stomach.*—Dr. J. Johnson lately stated to the London Medical Society, that in cases of simple irritation of the stomach, in which the organ did not bear well the presence of food, and digestion did not proceed in the usual manner, in conjunction with the use of small quantities only of the most nutritious and digestible food, nitrate of silver had proved to be one of the best remedies in lessening the irritation of the organ. He generally began with half a grain, given in the course of a day in three doses, increasing it, if necessary, to a grain and a half in the same period, but seldom exceeding this. In epilepsy, however, he had given as much as eight grains in 24 hours. Dr. J. also stated that in a sub-inflamed state of the mucous membrane about the fauces, in which there was thickening of the membrane, elongation of the uvula, with harassing cough and other symptoms, the nitrate of silver might be used with advantage. A plan which had been adopted in these cases with success, was to dip the sponge of a probang into a solution of the nitrate, and push it down the pharynx until it reached the upper part of the œsophagus. Highly irritable ulcers had also been relieved by this remedy. Dr. J. had never seen but one case in which the skin had been tinged blue by this medicine, which was a case of epilepsy, but in which the patient had continued the medicine six weeks beyond the time to which he was ordered to continue it. *Boston Jour.*

---

13. *Some Observations on the Use of the Hydriodate of Potass as a Remedy for Venereal and Cachectic Symptoms.*—There are few of our readers, who are not aware of the tendency to fashion that prevails in the exhibition of remedies. One year, calomel and opium are the rage—the next is consecrated to the glorification of colchicum—then every body must swallow strychnine—now, or at least lately, iodine was the mode. Some men fall more easily into the periodical prescribing of strange remedies than others do. A man of good common sense is loth to give up the use of a medicine he is acquainted with, for that of one he knows nothing about. If an old remedy answers in a certain case, he naturally asks himself "why should I change it? I know what it will do; but I do not know what effects this new drug may produce. When the remedies I am in the habit of employing fail, when the indications of treatment are not likely to be responded to by their familiar and well established properties, then I should be wrong not to try rational experiments with new medicines. Till then, you must excuse my rushing headlong, to the hazard of my patient and my own disgrace, into senseless though shewy trials of a pharmacopœia of novelties." Such is the calm rebuke, we may imagine a physician or surgeon possessed of sound judgment and experience, offering to the rash prescriber of new drugs.

There are many reasons why such drugs should be abused. The love of novelty is always operative, and constitutes at once a very useful and a very dangerous element in human nature. It is useful because it is the source of all great inventions—it is dangerous, when not moderated by caution and good sense, because it leads to perpetual change.

This passion for novelty is backed at the present day, by the spirit of journalism. The rapid phases of the weekly prints offer temptations to men of strong desires and weak minds, to publish incessantly any thing new, no matter whether it be philosophically true or not. It is painful to be obliged to admit that the medical press is in an unsound condition. Reputations are too often sacrificed from a spirit of personal hostility, and as openly awarded from motives of personal favoritism. The professional merits of a man are determined by a standard, decisive enough, but certainly not honest. Does he belong to a certain party? If he does, he is a second Hunter, or a Baillie, or a Cuvier—an admirable lecturer, although he stutters to snoring pupils—an excellent practitioner, although every body of discretion is glad to escape from his clutches. If he does not belong to the proper party, his ignorance and imbecility become immediately conspicuous.

Party rage was always deemed to have gone its utmost lengths in the political world, and in the political press. Of late years, that press has been distinguished on the whole for its ability and honesty. It is true, that extreme opinions have been advocated. Yet in the leading journals while political creeds are enforced with all the power of language, their columns are seldom polluted with gross ribaldry or disgusting personalities, never with deliberate untruths. It has been reserved for a scientific profession to exhibit in its literary appetite a keen relish for slander, and a taste for what is frequently neither more nor less than falsehood.

Journalism has encouraged the disposition to attract the profession, we will not say to humbug it, by positive assertions of the powers of new drugs. It is easy, as experience has proved, to get up a glittering array of cases, in which some newly-imported remedy has effected marvellous cures. The vulgar stare, and think that he must be a very clever fellow who has done all this. Gradually indeed the credulous are undeceived, and experience, after a season, shews how exaggerated the statements were. But the experimenter's purpose has been served. He has attracted notice, has been consulted in strange and in common cases, and as soon as the old bubble has fairly burst, he blows another. Such is a brief sketch of what has occurred on the professional stage There have been too many actors in the farce, but we do not allude in particular to any one. Yet conscious sensitiveness may induce this or that individual to imagine, that we point at him.

> When you mention vice or bribe,
> 'Tis so pat to all the tribe:—
> Each cries—"that was levelled at me."

In the present article, we merely intend adverting to the hydriodate of potass, a remedy which has been latterly used in many complaints of very opposite descriptions, but has been especially employed in the treatment of venereal symptoms. We shall make a chapter in the work of Mr. Judd, and a lecture by Mr. Wallace, of Dublin, the text on which we shall hang some comments.

We have already drawn attention to the exhibition of the hydriodate of potass in cases of syphilis, particularly in two notices of two papers by Dr. Williams.* We remarked that, on the whole, the evidence adduced was unsatisfactory, and that the actual powers of the hydriodate yet remained to be determined.

Mr. Judd's observations on the hydriodate are imperfect. They are rather anticipations of what it may do, than actual statements of what it has done. The facts he states are four in number, and all are instances of secondary symptoms. We shall give a sketch of them.

1. A man had connexion with a prostitute on the 17th of June, 1833. On the 20th he had urethritis and a pimple on the body of the penis. On the 22d, the pimple had become a sore, which healed on the 30th, and the urethritis ceased.

On the 27th of September, we find him with ecthyma, ulceration on the lower part of the tibia, an ulcer at the back of the throat, a gleet, and an excoriated glans penis. Under the employment of antimony, the warm bath, etc., followed by the infusions of gentian, the ecthyma decliued, the ulceration healed, and chocolate-colored stains, with œdematous ankles, and a few fresh flat pustules were left on the 28th of October. On the 1st of November there came out an eruption of lichen,

* One of those papers was published in the Medical Gazette—the other in the first number of the St. Thomas's Hospital Reports.

which assumed a pustular character, and faded. The patient grew fat, but was pale and bloated, and suffered from severe nocturnal pains in his shins, when, on the 12th of December, he was ordered—

*Potass. hydriod.* gr. v. *ter. die.*

By the 24th, he was quite free from pains, and was in good health. In February, he had, for a fortnight, effusions into the abdomen, which were removed by purgative diuretics; and by a report, dated July 12, we find that there had been no relapse.

2. On the 10th of November, 1832, a man had connexion with a common woman. On the 2nd of January, a slight sore commenced on the glans penis, for which he took five grains of blue pill twice daily. How long this was continued it is difficult to say, for the case displays the ordinary obscurity of Mr. Judd. On the 9th, bubo which did not suppurate. On the 12th, the sore healed. On the 11th of February, after exposure to wet and cold, a sore throat, succeeded by pains, and in April, by a discharge of pus and blood from the nostrils. On the 18th of May, he presented rupia. It would appear that mercury was persevered in till the 19th, a period of upwards of four months! He suffered, with slight intermissions, from severe pains in the forehead and temple, the bloody discharge, now sanious, continuing. On the 24th of August, he was ordered to rub in, with temporary benefit. On the 18th of September, an abscess formed over the right os nasi, where a large ulcer formed. On the 4th of October, there came pain and swelling of the left eyebrow, which was extremely indurated. Then followed an abscess in the lid, whence the probe passed to carious os frontis. On the 9th of November, periostitis of the left shin. From Aug. 30th to Nov. 11th, the mouth was kept sore. On the 19th, the protuberant part of the lid sloughed. On the 24th, a large thin shell of bone came away from the throat.

26th. "The left nostril has become ulcerated within, and excoriated without; every now and then yellow discharge concretes upon it, and the patient is excessively plagued by it. A bent probe passed in by one nostril goes through the septum and out at the other; the part, once the bridge, of his nose, has sunk upon the face so as to form a slightly curved hollow: he has a pallid, leaden hue of countenance, he loses strength, and his nocturnal pains continue."

Thus he went on, his health being much impaired, his sufferings great, the nocturnal pains severe, the ankles swelling, when, on the 15th of December, he was ordered *potass. hydrio.* gr. v. *ter die in aqua menth.*

To this was added a pint of new ale daily.

On the 21st, the nocturnal pains had almost ceased, and his health was better. On the 26th, the swelling of the forehead and eyelid had quite subsided. The ulcers soon after healed, and, in short, recovery took place in a highly satisfactory manner.

3. "In another secondary case, that of a young gentleman (the original disease not being contracted in England,) with eruptions on the skin, pains in the shins and effusion into the synovial membrane of the knee, I easily effected a cure by this same remedy. He hunts and gets wet, and although more than a year has elapsed, he has not experienced any return of his complaint."

4. "The hydriodate in the present month, in another instance, quickly removed an eruption and nocturnal pains; but the bubo remains to be healed. I am also trying the remedy upon a node, but have not continued its use long enough to ascertain how far it is efficacious in removing osseous dispositions."

Such are the effects, such the evidence adduced by Mr. Judd. When we examine it, we perceive that the cases in which the hydriodate was beneficial were of the cachectic character—cases to which mercury is notoriously, as a specific, inapplicable—cases, indeed, which are generally the result of the abuse of mercury. In the second case this was plainly the fact, and the unfortunate patient had good reason to complain of the treatment adopted in the first instance.

Let us return to Mr. Wallace.

This gentleman has tried the effects of the hydriodate in many cases of secondary symptoms. He has formerly, it would seem, employed other preparations of iodine, but latterly he has confined himself exclusively to the hydriodate, and for rather a singular reason. Most persons would use this or that form of a medicine because it answered better than another. The chief consideration, however, with Mr. Wallace would appear to be a speculative one; at all events, he puts his hypothesis in the front of the battle.

"In the first place," he says, "I believe, that whatever preparation of iodine you use, it is only in the state of hydriodic acid, or of a hydriodate, that it enters the system. I have never been able to detect free iodine in any part of the body, or in any of the excretions. If you administer iodine in its free state to a dog, and examine the contents of his stomach, after the lapse of a very short time, you will not be able to detect an atom of iodine in the free state. You will find that it has all been converted into hydriodic acid. On one occasion, Dr. O'Shaughnessy could detect no iodine in the matter discharged from the stomach of a dog to which he had given this substance fifteen minutes previously, but he found a large quantity of hydriodic acid. Are we not, from these facts, entitled to arrive at the conclusion that iodine, as I have said, enters the system only as an hydriodate or as hydriodic acid? If this be the case, it is quite useless to employ iodine in any other form, when it is our object to introduce it into the system."

We would observe that it by no means follows, because iodine is converted in the stomach in hydriodic acid, that therefore it is of no use giving iodine in any other form than that of hydriodic acid. This is as palpable a bull as ever gored Paddy. If Mr. Wallace could show that hydriodic acid was the only efficient form of the medicine, and that iodine exhibited in any other way would *not* be converted into hydriodic acid in the stomach, then his conclusion would logically follow from his premises. As it is, we have a true Hibernian non sequitur. Put it in the form of a syllogism, and see how it looks. Hydriodic acid is the only form that gets into the system—but iodine, introduced into the stomach, immediately becomes hydriodic acid—therefore, it is useless to give iodine.

The second reason which Mr. Wallace urges against any form of iodine but the hydriodate is a tangible one. He affirms first that iodine in its free state is very irritating to the stomach, the conversion into the hydriodic acid not being sufficiently rapid to prevent disturbance or even inflammation of the mucous membrane. And, secondly, he declares that the hydriodate of potass does not give rise to such injurious effects. This is the plain expression of a fact, and we have only to enquire if the fact is a true one.

We are disposed to go some way with Mr. Wallace, but not to the very end of his conclusion. We decidedly think that he overrates the mischievousness of the tincture of iodine, or of other forms of it besides the hydriodate. We have seen the tincture exhibited on a considerable scale, and we have given it freely ourselves. When the case was judiciously selected, and the medicine carefully managed, we have seen no bad effects whatever from its use. Perhaps there are few remedies which have been more extensively prescribed in London within the last few years, and though rash and ignorant people have no doubt abused it, still the experience of the best physicians and surgeons of the metropolis will certainly not be found to correspond with Mr. Wallace's. It is rather remarkable that when iodine was first employed for the relief of secondary symptoms, it was this very calumniated tincture, that effected those rather surprising cures which attracted the notice of the profession. It is a common practice to "give a dog a bad name," but, scientifically speaking, we may question its propriety.

While we defend the tincture of iodine against the unmerited obloquy heaped on it, we agree with Mr. Wallace that, on the whole, the hydriodate of potass is the preferable preparation. It is milder in its effects, less variable in its operation, and while it does not stimulate the intestinal mucous membrane so much, it appears to exert less of that peculiar depressing influence occasionally witnessed after the exhibition of the tincture. Common experience is seldom, in the long-run, false, in cases to the determination of which it is applicable. That experience has, in this country, given the general preference to the hydriodate of potass. It is probable that Mr. Wallace has too bad an opinion of the tincture of iodine, but in declaring the superiority of the hydriodate of potass, at all events in venereal affections, he echoes the prevailing sentiment.

Mr. Wallace dwells much on the presence of the hydriodate in the urine, and in some of the secretions. There is nothing in this to attract attention. It is only what occurs with hundreds of drugs as well as with articles of diet.

The effects which he has observed to follow the employment of the medicine may be briefly stated :—

1. Increase of appetite, strength, and spirits.

2. Increase of the excretions, and secretions ; of the urine—the perspiration—the saliva—increased action of the bowels. None of the effects are constant, and even the reverse of any one of them may happen.

3. Occasionally the hydriodate disagrees with the stomach, occasioning dyspepsia, heartburn, and a sort of sore throat. These accidents, Mr. Wallace informs us, are controllable by quinine.

4. Some patients have complained of watchfulness, and of a feeling in the head, approaching to headache. A purgative, and intermission of the medicine, have been necessary.

5. In two patients, who were over-dosed, there was sickness, soreness of throat, colicky pains, vomiting, and purging to a slight degree, frequent pulse, and exhaustion. These symptoms were relieved by laxatives.

6. Several patients have been attacked with a pleuritic pain. When bled, the blood was cupped.

7. On one occasion purpura decidedly resulted from it.

8. In a patient who took the hydriodate indiscreetly and excessively by his own direction, there occurred indigestion of the character already noticed, with severe headache, and a rapid and quivering pulse. "But the most remarkable of his complaints was a peculiar state of his eyes,—such a state as I never have seen before or since. The pupils were dilated, and both his eyes were in a state of incessant motion. These motions strongly resembled those of a child who has congenital cataract. He found himself quite unable to fix them on any object. He kept his hand constantly over them, as if to shade them from the light, yet he seemed to say that the light did not hurt them." He complained of constant headache. Soon after this, he was seized with paralysis on one side of the body, preceded by muscular tremblings. He continued in a dangerous state for some weeks, when he began to amend, and has a reasonable prospect of recovery.

Such is the substance of Mr. Wallace's remarks. They appear to us to be very just, although we think he is inclined to under-estimate the ill effects, and overrate the good that springs from the use of the hydriodate. Be that as it may, it is obviously a matter of importance to understand rightly the operation and the action of any remedy, particularly of one of so powerful a character as the one before us.

Our own experience, so far as it goes, and latterly we have employed the hydriodate in many cases, agrees very nearly with that of Mr. Wallace. The experience of a hospital is frequently not borne out in private practice, the constitutions of the patients in one and the other presenting material differences. This may account for our finding the hydriodate disagree much more than Mr. Wallace seems to have done. The patients to whom we have given it have been mostly private patients—persons in the respectable classes in London. The subjects of his observations have been chiefly in and out patients of the infirmaries to which he is attached. We may well conceive that the stomach of a Patlander, accustomed to regale on potatoes and whiskey, will bear stronger stuff in the way of physic, than will that of an English gentleman or tradesman. Every one accustomed to hospital and private practice in London, is aware of the difference of dose necessary in the administration of many medicines, and of the difference of effects observed in their patients in the former and the latter.

Up to a certain point, the hydriodate of potass, like the iodine itself, appears to be a stimulant, if not a tonic. Certain it is, that it quickens the pulse, often increases the appetite and strength, will even occasion embonpoint, and excites more or less the whole system. It resembles in this respect arsenic, which, it is well known, will operate as a tonic, in certain doses, and in certain cases.

Yet the hydriodate cannot be deemed a mere tonic. The medicines of that class exhibit, on the whole, very different effects. If we watch, for example, the operation of bark, we find that it excites the action of the heart, and of the capillaries, increases the strength, and invigorates the system. If pushed to a certain point, the bad effects that follow are just what might be supposed to result from an excess of the same sort of action. Too much blood is made, and it is circulated with too much force—sanguineous congestions or local inflammations ensue—the whole system is oppressed and overloaded—but none of that peculiar depression occurs, which marks the influence of a poisonous agent, exerted on the nervous system, or displayed through its medium.

To call arsenic a tonic, if by tonic is to be understood a remedy like bark, is clearly an abuse of terms. Arsenic, in appropriate doses, will at first quicken the action of the heart, and excite the system much in the same way that a tonic does. Acting thus, it will even cure diseases for which bark is notoriously efficacious—neuralgia, or intermittent fever. But here the analogy abruptly stops. If the arsenic is still given, or if its dose is increased, a new train of symptoms are suddenly developed. Either inflammation of the gastro-intestinal mucous membrane supervenes, or a prostration occurs, that is evidently the result of a powerful poisonous agent. We have seen arsenic given for diseases of the tongue, agree very well for a certain time, and all at once the patient has been seized with tremblings, rapid and weak pulse, and deadly exhaustion. One patient died in this condition, and on dissection, nothing decisive was found. There was a slight redness of the gastric mucous membrane, and that was all.

There appears to us to be a greater analogy between the action of iodine and arsenic, than between that of iodine and any other medicine. We say analogy, for their specific effects are materially different.

Iodine, at first, like arsenic, stimulates the system, and excites the action of the heart and of the capillaries.* If carried too far, its effects are not simply, like those of quina, excess of stimulation, but, again like those of arsenic; poisonous and specific. The gastro-intestinal mucous membrane may suffer, or it may not. That is rather a local and accidental consequence. But whether it does or not, peculiar symptoms ensue—nervous agitation—rapidity and weakness of the heart's action—muscular tremblings and debility—general depression—and even death. We have seen all these effects, short of actual destruction, from the exhibition of iodine, both for cachexia and for malignant growths.

The hydriodate of potass differs from iodine in possessing more of the simply stimulating, and less of the peculiar and poisonous property. It is a difference, however, of degree and not of kind. The cases related by Mr. Wallace, and some that we could bring forward, are sufficient to show that, although it may be given with more freedom than free iodine, the same description of bad effects must be apprehended and guarded against. We need not fear them so much, but we must not forget that they *may* come.

Practically speaking, the injurious consequences of the hydriodate are rather of the immediate than remote description—they flow from its primary and stimulating qualities; not from its consecutive depressing ones. The mucous membrane of the stomach is irritated and excited, and secretes more acid, and the irritation extends to the œsophagus and pharynx. We have found uneasiness of the stomach, pyrosis, heartburn, and that peculiar constriction and heat in the throat alluded to by Mr. Wallace, very commonly produced by a continuance in the employment of the medicine. Occasionally we see still more distinctly the effects of the stimulant property of the hydriodate. Inflammation of the pleura, headache dependent on cerebral congestion, or inflammation of the gastro-intestinal mucous membrane, may ensue. It is not at all uncommon for persons who are taking this medicine to be attacked with heat of skin, headache, full pulse, loaded tongue, in short, with decided pyrexia. Probably, if the remedy were continued under these circumstances, either some positive local inflammation would arise, or the poisonous effects of the hydriodate would be developed.

We think, then, there can be little doubt that the sensible effects of the hydriodate of potass are in the first instance of a stimulating and a tonic character. We say sensible effects, for there may be, and there probably are others of a more refined and subtle description, which not being shown by any tangible alterations of the functions, are not so readily appreciated.

---

* When we talk of the action of the capillaries, we do not wish to advocate any hypothetical notions of their functions. We use the term for the want of a better, and refer merely to the operations of the capillary system, exhalation and secretion and absorption, without troubling ourselves about their causes.

Were we to believe some authors, the hydriodate is a medicine of great value in all secondary venereal symptoms. This does not agree with what we have seen, nor is it likely a priori to be true. Few things can be more opposed in all essential features, than a secondary papular or tubercular eruption, and the cachetic rupia or ecthyma. The affection, in the first instance, may be the direct and simple consequence of the syphilitic virus, the constitution of the patient is good, the symptoms are of a sthenic character, and mercury, if properly exhibited, effects a cure. In rupia or ecthyma all bespeaks cachexia. The vesicles or pustules form scabs, foul ulcers, or become phagedænic or sloughing sores—the periosteum or the bones may be affected—the patient is emaciated—debility is evident—and mercury, the abuse of which has usually caused the symptoms, almost always aggravates them. It is not likely, we say a priori, that any one remedy should suit equally such opposite states. It is not consistent with our common experience of medicines or of maladies, nor does it agree with what we have observed of the effects of the hydriodate itself.

The venereal cases in which we have seen most benefit from the hydriodate, have been chiefly those of a cachetic character—cases in which repeated courses of mercury have been given, and in which the patient either does nor require or will not bear that remedy. There are usually pustular or vesicular eruptions, producing scabs and unhealthy, sloughing, or creeping ulcers—ulceration of the throat of a character similar to that upon the skin—nodes, caries of the bones—chronic inflammation of the testis and the cord. Such is the kind of case in which the hydriodate appears, upon the whole, to do most good. It is frequently surprising to see how rapidly, and even permanently, the patient will recover. He may have gone on for months, or even for years, with only temporary and delusive periods of amendment, when a course of the hydriodate may set him at once and effectually upon his legs.

We lately attended a gentleman with Mr. Davis, an intelligent surgeon of Hertford. The patient had been ill for two or three years. When we saw him, there was considerable tumefaction of the alæ nasi, with redness of the integument, and discharge from the nostrils. There were several cachectic ulcerations on the skin—ulceration of the soft palate and pharynx—emaciation—and general ill health. We prescribed sarsaparilla and some other remedies, and the patient mended a little. But portions of diseased bone came away from the nose, and a piece, apparently of the horizontal process of the palate bone, was discharged through an ulcerated aperture in the front of the velum. We now put the patient on the hydriodate. The result was most gratifying. All the symptoms very speedily subsided, and the gentleman has been left with a small, round, circular aperture in the palate, productive of too little inconvenience to require the application of the suture.

We must own, that although occasionally the hydriodate has effected both rapid and permanent benefit, it has not in the majority of instances done so. It is the character of the cachectic disease we have mentioned, to return again and again, and to exhibit repeated vicissitudes of improvement and relapse. It is indeed a singular complaint, and one well calculated to illustrate the doctrines of the humoral pathology. A patient is attacked with an eruption of rupia or ecthyma, and the vesicles or pustules ulcerate or slough—at the same time, perhaps, ulceration of the throat appears or spreads, or sloughing invades the mucous membrane of the fauces or the nares. After a time these symptoms become mitigated—the sloughing is arrested—the ulcerations may heal—and the patient may even seem to be restored to health. But the recovery is deceptive. Suddenly, he is again seized with the whole or with a part of his former symptoms, which again exhibit the same successive phases of aggravation, arrest, and amendment or apparent cure. This scene may be, and generally is repeated many times, and the event depends on the treatment employed, and on the constitution of the patient. If both are bad, the attack grows more and more serious, or rather they leave him less and less able to sustain them. The bones may or may not become affected, but, whatever be the character of the specific symptoms, it is not from them, per se, that death usually results. Accidental circumstances lead to disease of some vital organ, and, in the majority of instances, either phthisis, or fever ending in ulcerations of the mucous membrane of the bowels, terminates the case.

It could hardly be expected that any remedy would at once cut short a malady of such a character. It is clearly a constitutional disease, and like scrofula, depends on too general an alteration of the system to be at once put out. Such anticipations are supported by experience, and it is seldom indeed that a case of any severity is quickly cured. Of course there are differences of degree in this, as in all other complaints, but we repeat that we must not anticipate an off-hand *and* permanent recovery from any system of management, undoubtedly not from any single drug.

In many of these cases the hydriodate agrees remarkably well. Given at the proper time, seems to arrest the phagedænic action, promote the cicatrization of the sores, and re-establish the health of the patient in a very remarkable degree. But many other remedies do the same thing. Sarsaparilla—the mineral acids—tonics—sometimes small doses of mercury, produce in these cases more or less of the same effects as the hydriodate. In short, it is preposterous to term this medicine a specific for this complaint, for though it sometimes answers better than any of the others we have mentioned, it not unfrequently does not answer so well. It is an addition, and a valuable addition to our means of treatment—it gives us one other medicine to ring the changes with, and that is the most that can fairly be said of it. We may mention a case which is sufficiently illustrative of these remarks.

*Case.*—A respectable tradesman, after a long and injudicious course of mercury for a venereal sore, was attacked with extensive ulceration of the fauces, and with a phagedænic ulceration on the left arm, which succeeded a vesicle that scabbed. A variety of means were tried. Sometimes he was better, sometimes he became worse. He was put on the hydriodate in full doses, with almost instantaneous benefit. The ulcer of the throat almost healed. Again it broke out, and again the hydriodate effected the greatest benefit. But still these relapses occurred, and now, at the end of two years and a half from the first appearance of the ulceration in the throat, the patient is in a very precarious condition. The fauces are covered with a slouthy ulceration. A portion of the lateral and nearly all the vertical cartilage of the nose have been destroyed—and gastro-enteritis following the *cautious* employment of the hydriodate nearly proved fatal about a month ago. In each attack the medicine has appeared to be of signal service, yet two years and a half have passed, and the individual is not materially benefitted, but rather the reverse.

We could mention many cases of this description, and we can assure our readers that there is not a greater error than to suppose that, because the hydriodate is of temporary, it is always of permanent service. It is a remedy which should be employed with judgment, caution, and that distrust which *must* spring from an acquaintance with the peculiar character of the disease.

Not unfrequently, it is either of no service at all, or it positively disagrees.

We have at present under our care a gentleman, whose case has been in some respects a singular one. Having had, to his knowledge, neither sore nor urethral discharge, he was attacked with yellow ulceration of the throat, and what looked precisely like the venereal tubercle. He consulted a surgeon, who felt unwilling to order mercury, and prescribed the tincture of iodine. The ulceration quickly healed, and the eruption appeared to decline. But soon both re-appeared. We saw him about nine months after the commencement of the ulceration of the throat. Both tonsils were then the seat of sloughy ulcers, and there were several spots of rupia on the skin. He had also secondary ulcerations at the verge of the anus, which were exquisitely painful. We ordered a course of calomel and opium, under which the ulcerations of the rectum and of the throat healed, and have never since returned. But the rupia spots ulcerated, and at intervals fresh sloughy ulcerations have appeared on differant parts of the body, accompanied latterly with nodes. We have repeatedly tried the hydriodate in this case. After a few days, it occasions heart-burn, pain at the epigastrium, pyrexia, and a more sloughy state of the sores. To these it has never been of service, and the patient has been always made ill by it.

In cachectic cases of a more obscure character, and totally unconnected with syphillis or with the abuse of mercury, the hydriodate occasionally does wonders.

*Case.*—A gentleman was seized with what appeared to be pleurisy. He was bled to a great, indeed, to an improper extent. He had previously been a free liver, and the depletion reduced him in such a manner, that he never properly rallied. Under these circumstances, he was attacked with violent pains in the bones—he was sallow, emaciated, and had all the appearance of a man broken down with the venereal cachexia. He was under the care of the most eminent physicians at Cheltenham, but got no better, or not materially so. He came to London, and consulted an eminent surgeon, who prescribed the hydriodate. The pains in a week or two disappeared, and he rapidly recovered. Whether the cure will be permanent, it is of course impossible to say.

The hydriodate has been applied to the treatment of other venereal affections, besides the cachexiæ we have spoken of. In the present article, we confine ourselves to what we have personally witnessed, and shall, therefore, state only the results of our own observation.

1. We have not tried it in cases of primary syphilis, nor do we *imagine*, from what we know of its operation, that it will be of service. Perhaps, in some cases of primary phagedænic ulceration, it may be useful.

2. Nor, from what we have witnessed, should we conceive that it is applicable to the pure syphilitic eruptions—the scaly, or the tubercular. These eruptions are so much under the control of mercury, and its action is so different from that of the hydriodate, that it seems both unnecessary and unreasonable to employ, or to anticipate much from the employment of the latter.

3. It has been lauded in cases of papular eruption. But we should pause before we attach much importance to its use in *them*. Lichen is so fugitive in its character, that it is difficult to determine the effects of remedies upon it—at all events, there is an obvious source of fallacy.

4. We have tried the hydriodate in some of the more vague secondary venereal symptoms.

*a*. In a case of enlarged tonsils, covered with white superficial ulceration, and connected with vaginal discharge and condyloma, it was of no benefit.

*b*. A gentleman had sores, which were said in Paris to be excoriations, and were treated in England as syphilitic. He seems to have taken a good deal of mercury. His health had been impaired by previous dysentery, contracted in Syria, while travelling for amusement. Two years afterwards we saw him. He was liable to frequent irruptions of small superficial sores on the lips and tongue, and had psoriasis palmaria. We have tried the hydriodate three times in this gentleman. Each time it occasioned dyspepsia, and did no good to the complaint. In another similar case, we used the medicine with the same want of success. In a third case, it was of use, but the disorder was getting well when we ordered the hydriodate to be taken.

Such are the results of our own experience. We have employed the hydriodate in other cases, but the results were not sufficiently distinct to induce us to allude to them. On the whole, we are disposed to coincide with those who have found the hydriodate answer best in cachectic cases. But we think that, even in these, it is uncertain, and that it has been overrated. It is another string, and not a bad one, to a bow that has several already, but requires more.

The most usual mode of exhibiting the hydriodate is in the state of solution in camphor mixture. It is nauseous in that form. Perhaps the pleasantest formula is the succeeding, or one like it.

℞ Aquæ Florum Aurantii, ℨiij.

Aquæ Distill. - - - - ℥j.

Syrupi Aurantii - - - ℥j.

Potass. Hydriodatis - - gr. v. ad x.

M. ft Haustus ter die bibendus.

We have found that, in cachectic cases, it is usually better to conjoin the hydriodate with small quantities of sarsaparilla. Either the fluid vehicle of the hydriodate may be sweetened with a drachm or two of the syrup of sarsaparilla; or the latter, in the most appropriate form, may be taken a little after the dose of the hydriodate.

In private practice, patients will seldom bear more than ten or twelve grains of the hydriodate thrice daily. It is better to begin with small doses, and gradually increase them. We may commence with five grains twice in the day, and augment the proportion to the requisite amount.—*Med. Chirur. Rev.*

